# Projected Time for the Elimination of Cervical Cancer Under Various Intervention Scenarios: Age-Period-Cohort Macrosimulation Study

**DOI:** 10.2196/46360

**Published:** 2024-04-18

**Authors:** Yi-Chu Chen, Yun-Yuan Chen, Shih-Yung Su, Jing-Rong Jhuang, Chun-Ju Chiang, Ya-Wen Yang, Li-Ju Lin, Chao-Chun Wu, Wen-Chung Lee

**Affiliations:** 1 Institute of Epidemiology and Preventive Medicine College of Public Health National Taiwan University Taipei Taiwan; 2 Head Office Taiwan Blood Services Foundation Taipei Taiwan; 3 Master Program in Statistics National Taiwan University Taipei Taiwan; 4 Institute of Statistical Science Academia Sinica Taipei Taiwan; 5 Taiwan Cancer Registry Taipei city Taiwan; 6 Health Promotion Administration Ministry of Health and Welfare Taipei Taiwan; 7 Institute of Health Data Analytics College of Public Health National Taiwan University Taipei Taiwan

**Keywords:** age-period-cohort model, population attributable fraction, macrosimulation, cancer screening, human papillomavirus, HPV, cervical cancer, intervention, women, cervical screening, public health intervention

## Abstract

**Background:**

The World Health Organization aims for the global elimination of cervical cancer, necessitating modeling studies to forecast long-term outcomes.

**Objective:**

This paper introduces a macrosimulation framework using age-period-cohort modeling and population attributable fractions to predict the timeline for eliminating cervical cancer in Taiwan.

**Methods:**

Data for cervical cancer cases from 1997 to 2016 were obtained from the Taiwan Cancer Registry. Future incidence rates under the current approach and various intervention strategies, such as scaled-up screening (cytology based or human papillomavirus [HPV] based) and HPV vaccination, were projected.

**Results:**

Our projections indicate that Taiwan could eliminate cervical cancer by 2050 with either 70% compliance in cytology-based or HPV-based screening or 90% HPV vaccination coverage. The years projected for elimination are 2047 and 2035 for cytology-based and HPV-based screening, respectively; 2050 for vaccination alone; and 2038 and 2033 for combined screening and vaccination approaches.

**Conclusions:**

The age-period-cohort macrosimulation framework offers a valuable policy analysis tool for cervical cancer control. Our findings can inform strategies in other high-incidence countries, serving as a benchmark for global efforts to eliminate the disease.

## Introduction

Cervical cancer remains the fourth most common cancer in women worldwide [1]. The World Health Organization (WHO) [2] called for the global elimination of cervical cancer; elimination is defined as an incidence of fewer than 4 cases per 100,000 women-years. Eliminating cervical cancer requires scaling up the human papillomavirus (HPV) vaccination of girls and cervical screening [3].

Randomized trials and follow-up studies are valuable for evaluating the short-term and medium- to long-term impacts of public health intervention policies [4,5]. In contrast, modeling studies offer the ability to estimate and forecast effects over an extended timeframe and can also simulate outcomes across a range of future scenarios [6]. Numerous microsimulation modeling studies have been conducted to assess the necessary time frame and intervention strategies for the elimination of cervical cancer [3,7-14]. These studies rely on numerous assumptions, such as oncogenic potentials, infection dynamics and immunity of HPV, and the natural history of cervical cancer. They require many parameters that are difficult to obtain.

A better alternative is to use macrosimulation modeling. From a macroscopic perspective, disease incidence rates are primarily influenced by 3 temporal factors: age, period, and cohort. The age-period-cohort (APC) model, which accounts for these factors, has been recently used to project future disease burdens [15-19]. This methodology can also estimate future incidence rates of cervical cancer under existing conditions, known as the status quo. In a hypothetical “what if” scenario involving a public health intervention, the baseline incidence rate would be reduced by a certain fraction—captured by the population attributable fraction (PAF) associated with the intervention. By combining the APC model’s baseline estimates with the PAF to factor in reductions from interventions, we can effectively forecast future cervical cancer incidence rates under specific public health programs.

Well-organized cytology-based cervical screening programs have effectively reduced the incidence rates of cervical cancer in many countries [20-23]. Since 1995, Taiwan’s Health Promotion Administration has offered organized cytology-based screenings for women aged 30 years and older. Consequently, the age-standardized incidence rate of invasive cervical cancer in Taiwan sharply declined from 28.0 per 100,000 woman-years in 1997 to 8.2 in 2016. Despite this progress, the incidence rate in Taiwan has yet to meet the criteria for the elimination of cervical cancer. A more effective form of HPV-based screening, which has shown to be superior to cytology-based methods [24], may soon be adopted for mass screening. Projecting future trends in cervical cancer incidence under new approaches such as HPV-based screening has been challenging using microsimulation models. However, these projections may be more feasible through macrosimulation techniques.

In this study, macrosimulation was used, combining APC modeling with PAF calculation, to estimate when cervical cancer could be eliminated in Taiwan. We explored the potential to expedite this timeline by implementing more extensive population-based cervical cancer screening (either cytology or HPV based) and enhancing HPV vaccination coverage.

## Methods

### Data Source

In this study, we collected data on cervical cancer cases diagnosed between 1997 and 2016 using the *International Classification of Diseases for Oncology, Third Edition* code C53. Our extensive data set, which includes population information, originated from the Taiwan Cancer Registry—a meticulously maintained nationwide system established by the Ministry of Health and Welfare. The registry diligently captures and synthesizes information from patients who are newly diagnosed with malignant cancer in hospitals with 50 or more beds in Taiwan, a country with a population size of approximately 23 million. The data quality and completeness of the Taiwan Cancer Registry database have consistently adhered to standards of excellence. Specifically, the completeness is 98.4% (14,833/218,239); the percentage of cases with death certificate only is 0.9% (1079/118,583); the mortality versus incidence ratio is 45.1% (202.89/449.59); the percentage of morphological verification is 93% (109,273/117,504) for all sites combined and 97.6% (103,812/106,423) for all sites excluding the liver; and the data timeliness is 14 months. These statistics demonstrate that the Taiwan Cancer Registry is one of the highest-quality cancer registries in the world [25-27]. The world standard population (WHO 2000 [28]) proportions were used to calculate age-standardized incidence rates. The projected elimination year was defined as the first year when the age-standardized incidence rate would fall below 4 cases per 100,000 women-years [8].

### Macrosimulation

The APC models were used to project future cervical cancer incidence rates under the status quo. Due to the scarcity of cases in patients younger than 30 years of age, this age group was excluded from the APC modeling. Instead, the average incidence rate between 1997 and 2016 was used as the projected rate for women younger than 30 years of age. This approach likely makes our future decline estimates slightly conservative. However, the impact is minimal as the incidence rate for this age group contributes only a small fraction to the overall rate. For women aged 30 years and older, the projections are outlined below. First, an ensemble of 265 APC models was constructed using data from 1997 to 2006 as the training set. The APC models were then applied to project incidence rates from 2007 to 2016 as the validation set. These projections underwent year-on-year attenuation adjustments ranging from 0% and 5% to 100%, resulting in 5565 different projection sets. The symmetric mean absolute percentage error (SMAPE) was used to quantify the prediction error for each projection model, and the model with the lowest SMAPE was selected. Finally, the selected model, fine-tuned with all available data from 1997 to 2016, was used to project future incidence rates up to the year 2050. These projections incorporated the chosen attenuation factor. The future projected incidence rates were determined by calculating age-standardized rates across all age groups. For those younger than 30 years of age, the average incidence rate from 1997 to 2016 was used. For individuals aged 30 years and older, the rates forecasted by the selected APC model were used.

Next, we calculated the PAFs for various scenarios of cervical cancer screening and HPV vaccination. The PAF in this study was defined as the proportionate reduction in the incidence rate of cervical cancer due to a specific intervention program, as in the following equation [29]:



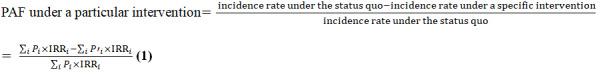



where IRR*_i_* is the incidence rate ratio between the *i*th level of the screening or vaccination variable and the reference level (*i*=1) of no screening and no vaccination, and *P_i_* and *P*ʹ*_i_* are the proportions of women in the *i*th level of the screening or vaccination variable under the status quo and the specific interventions, respectively.

The incidence rate ratios of cytology-based screening, HPV-based screening, and HPV vaccination were based on the studies of Chen et al [30], Ronco et al [24], and Lei et al [5]. We assume that HPV vaccination administered to 13-year-old girls affords them lifetime effectiveness against cervical cancer [31]. HPV-based screening may have a higher false positive rate, so it is suggested that screening be performed once every 5-10 years [24]. Currently, only cytology-based screening is available in Taiwan, and HPV-based screening still needs to be implemented. We also assume that the incidence rate ratios of a joint program involving both interventions are the products of 2 programs involving only the respective intervention (Table S1 in Multimedia Appendix 1). In 2016, the proportion of women with cytology-based screening more than twice in 6 years was 43.5% (data provided by Taiwan’s Health Promotion Administration), and a negligible proportion of women had received HPV vaccination in Taiwan (Table S2 in Multimedia Appendix 1).

We assume a gradual increase from 2023 to 2030 in the proportion of women undergoing cytology-based screening more than twice within 6 years from the current 43.5% to 70%. We also assume a new intervention plan from 2023 that involves switching from cytology-based screening twice within 6 years to HPV-based screening. The goal is to increase the overall screening proportion from 43.5% to 70% by 2030. We set an intervention scenario to achieve HPV vaccination coverage of 90% since 2018 (the Health Promotion Administration in Taiwan has offered HPV vaccination for 13-year-old girls since 2018). Furthermore, the potential impact of combining cytology-based and HPV-based screening with HPV vaccination in 2 different scenarios was evaluated. For simplicity, we assume that compliance with cytology-based screening, HPV-based screening, and receipt of the HPV vaccination are 3 independent events. The referral rate for positive cervical cytology results, facilitated by community nurses, exceeds 90%. Additionally, Taiwan’s health care system offers almost universal coverage, making effective cervical disease management an integral part of the existing health care infrastructure. As such, this study did not specifically factor in the concept of effective management.

We used equation 1 to calculate the PAFs for all 6 intervention scenarios considered in this study. The incidence rate under a specific intervention was then calculated using the following equation:

Incidence rate under a specific intervention = incidence rate under the status quo × (1 – PAF under a specific intervention) **(2)**

Data management and analyses were performed using SAS statistical software (version 9.4; SAS Institute Inc).

### Ethical Considerations

The study was based solely on deidentified aggregate data, without access to individual records. This study protocol was approved by the National Taiwan University Research Ethics Committee (NTU-REC 202101HM030) and the data release review board of the Health Promotion Administration, Ministry of Health and Welfare in Taiwan. All methods were performed in accordance with the relevant guidelines and regulations. In addition, the National Taiwan University Research Ethics Committee waived the requirement for informed consent due to the lack of personal information and secondary data in the study.

## Results

The selected projection model under the status quo was a polynomial APC model with a log link function and 55% attenuation (SMAPE=6.1%). Figure S1 in Multimedia Appendix 1 presents the observed and model-fitted age-standardized (WHO 2000 standard population [28]) cervical cancer incidence rates from 1997 to 2016 and the projections from 2017 to 2050. A declining trend in cervical cancer incidence rate was observed in the 20-year study period. In 2016, under the status quo of cytology-based screening compliance of 43.5% and no HPV vaccination, the projected cervical cancer incidence rate will not fall below 4 new cases per 100,000 women-years by 2050.

**Figure 1:**
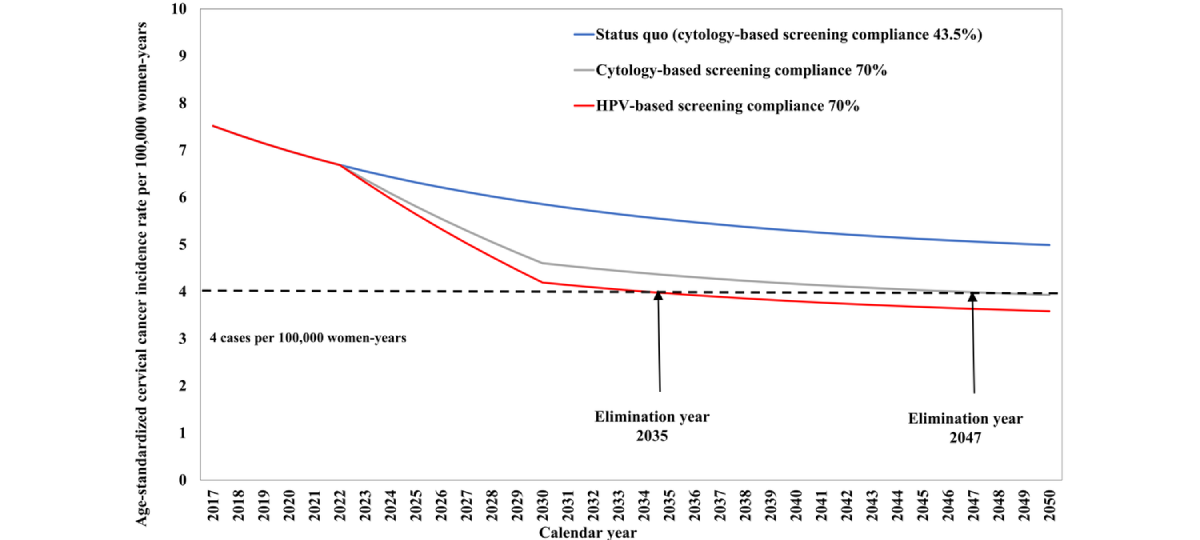
Future projections of age-standardized cervical cancer incidence rate per 100,000 person-years under the status quo, cytology-based, and HPV-based screening. HPV: human papillomavirus.

Figure 1 shows the expected incidence rates of cervical cancer from 2023 to 2030 under 3 scenarios: the current situation, if adherence to cytology-based screening increases to 70%, and if adherence to HPV-based screening rises to 70%. The projection indicates that cervical cancer in Taiwan will not reach the goal of elimination by 2050 if cytology-based screening compliance remains at the current level of 43.5%. However, if cytology-based and HPV-based screening compliance are increased to 70%, cervical cancer elimination can be achieved by 2047 and 2035, respectively.

**Figure 2:**
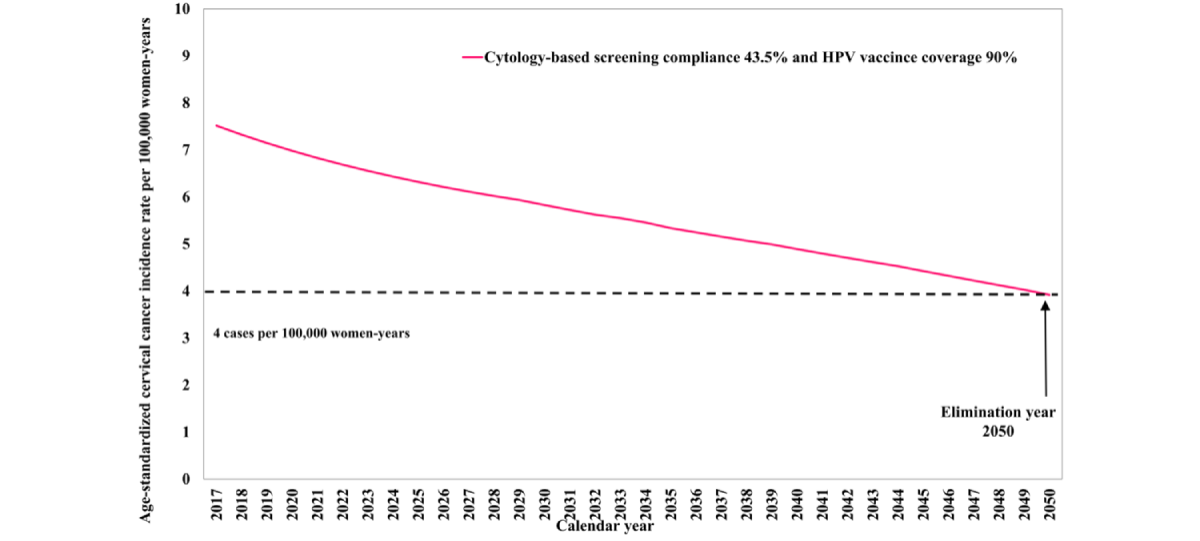
Future projections of age-standardized cervical cancer incidence rate per 100,000 person-years under HPV vaccination. HPV: human papillomavirus.

**Figure 3:**
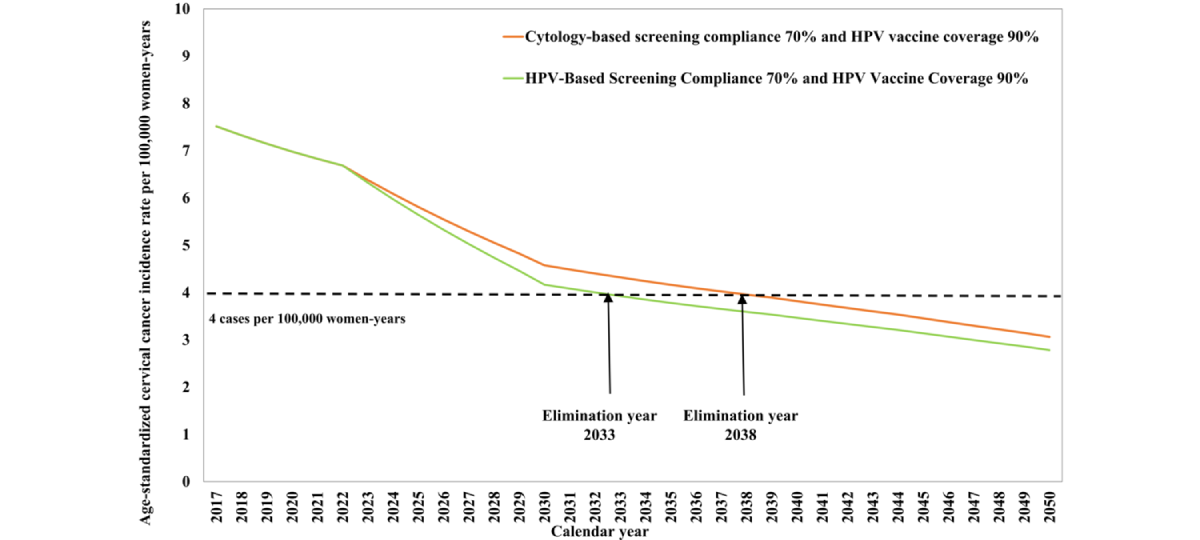
Future projections of age-standardized cervical cancer incidence rate per 100,000 person-years under scenarios of joint cytology-based and HPV-based screening with HPV vaccination. HPV: human papillomavirus.

Figure 2 shows the projected cervical cancer incidence rate with an HPV vaccination coverage of 90%. An HPV vaccination coverage of 90% will eliminate cervical cancer by 2050. Figure 3 presents the projected cervical cancer incidence rates if cytology-based or HPV-based screening is applied (compliance raised to 70%) in conjunction with HPV vaccination (90% coverage). Both joint interventions will help to achieve cervical cancer elimination before 2050 and at an earlier year (2038 and 2033, respectively).

The years of elimination (if before 2050) for the various scenarios are shown in Table 1. For comparison, the same table also shows the results when the Segi standard population [32] was used for age standardization. The time to elimination was expedited by a few years when the Segi standard was used.

**Table 1:** Projected elimination years^a^ under the status quo and various intervention scenarios, when the rates are age-standardized to World Health Organization 2000’s standard population and the Segi standard population.

Scenario	Projected elimination year (Segi standard population)
Status quo (cytology-based screening compliance 43.5% and no HPV^b^ vaccination)	—^c^
Scenario 1 (cytology-based screening compliance 70% and no HPV vaccination)	2047 (2035)
Scenario 2 (HPV-based screening compliance 70% and no HPV vaccination)	2035 (2030)
Scenario 3 (cytology-based screening compliance 43.5% and HPV vaccine coverage 90%)	2050 (2046)
Scenario 4 (cytology-based screening compliance 70% and HPV vaccine coverage 90%)	2038 (2033)
Scenario 5 (HPV-based screening compliance 70% and HPV vaccine coverage 90%)	2033 (2030)

^a^The first year when the projected age-standardized cervical cancer incidence rate falls below 4 cases per 100,000 women-years.

^b^HPV: human papillomavirus.

^c^The projected age-standardized cervical cancer incidence rate would not fall below 4 cases per 1000,000 women-years before 2050.

## Discussion

### Principal Findings

In summary, our macrosimulation analysis projected that Taiwan could eliminate cervical cancer by 2050 through 70% compliance with screening (cytology based or HPV based) or 90% coverage of HPV vaccination. Specifically, the projected elimination years are 2047 or 2035 for screening (cytology based or HPV based, respectively), 2050 for vaccination, and 2038 or 2033 for a combination of both screening and vaccination. This study confirms earlier microsimulation findings that increased screening can fast-track cervical cancer elimination [3,7-14]. Our macrosimulation methodology offers both adaptability and ease of implementation, as demonstrated by the SAS code in Multimedia Appendix 2. Unique to Taiwan is its high HPV vaccine coverage, a legacy of successful vaccine campaigns, facilitated by extensive health care infrastructure [33]. In stark contrast, Japan saw HPV vaccine coverage collapse from 70% to nearly 0% between 2013 and 2019 due to a crisis [14].

Cytology-based screening is crucial for cervical cancer management [34]. It is recommended that women aged 30 years and older should undergo cytology-based screening at least once every 3 years (effective screening). Historically, cervical cancer incidence has steadily decreased because of opportunistic and organized cytology-based screening [35]. From 1974 to 1984, Taiwan launched an opportunistic cytology-based screening program for cervical cancer, facilitated through partnerships between the cancer society and gynecology and obstetrics clinics [36]. A major turning point came in 1995 when Taiwan’s Health Promotion Administration set up an organized cytology-based screening initiative targeting women aged 30 years and older. This represented a significant advancement in Taiwan’s efforts to tackle cervical cancer. Consequently, the age-standardized incidence rate for invasive cervical cancer in Taiwan dropped from 28.0 per 100,000 woman-years in 1997 to 8.2 per 100,000 woman-years in 2016. This reduction marked a shift from Taiwan being a high-risk region to becoming a low-to-medium-risk area for the disease (the figure was presented in Figure S2 in Multimedia Appendix 1).

However, the need for improved compliance with effective screening is a global issue, and Taiwan is no exception [37]. Although approximately 82% of Taiwanese women have undergone at least one screening since 1995, the overall effectiveness of screening implementation remains below the desired level. The participation rate for triennial screenings, aimed at women aged 30 to 69 years, is only around 54%. Moreover, participation drops even further among women aged 69 years and older. These data underscore the pressing need for improved outreach and accessibility to achieve more comprehensive and impactful screening initiatives [38]. Various strategies have been adopted to promote screening, including educational interventions, physician reminders, incentive programs, mass media campaigns, outreach to community members, and leveraging community health workers [39-41]. Despite all these efforts, the compliance rate of effective screening in 2016 was only 43.5% in Taiwan. Our analysis indicates that cervical cancer in Taiwan can be eliminated in 2047 only if there is 70% compliance with cytology-based screening. To enhance the effectiveness of screening, strategies could include distributing informative pamphlets to schoolchildren to share with adult women in their families; authorizing self-collected HPV screening kits (Taiwan currently only approves the use of clinical-based HPV screening kits); and targeting older, previously unscreened and unvaccinated groups.

Cytology-based screening as a primary mode of cervical cancer screening has been gradually replaced by HPV-based screening, which has 70% greater protection against invasive cervical cancer [24]. HPV testing recommended by WHO may increase engagement in cervical cancer screening programs [42]. Taiwan has been implementing cytology-based screening for more than 30 years; however, compliance still needs to be improved. The Health Promotion Administration in Taiwan is also considering implementing more effective HPV-based screening. This study demonstrated that HPV-based screening could achieve the goal of eliminating cervical cancer more swiftly than cytology-based screening, given the same conditions. Taiwan has successfully decreased the incidence rate of cervical cancer through an organized cytology-based screening program. It is now considering the implementation of HPV-based screening to achieve the goal of elimination faster. This approach can be used as a model for other countries.

If HPV vaccine efficacy wanes, it may alter cervical cancer prevention and screening protocols [43]. However, long-term studies since the vaccine’s 2006 introduction show sustained high antibody levels, suggesting it could offer near-lifelong cervical cancer protection [44-50]. However, multiple factors hinder the implementation and scaling-up of HPV vaccination, such as vaccine supply shortage [51,52], budgetary constraints [53], and hesitancy due to vaccine-related side effects [54]. Globally, only a few high-income countries have offered the HPV vaccine for the target age group with a coverage rate above the WHO-recommended threshold of 90% [51,55]. By comparison, the HPV vaccination program introduced in Taiwan in 2018 has been relatively successful; the coverage rates were 76.8% in 2018 and 86.9% in 2019, respectively. It is very likely to further increase this rate to 90% in 2022 (scenario 3 in this study); if this happens, we project that cervical cancer in Taiwan can be eliminated in 2050. The effect of HPV vaccination (on 13-year-old girls) can only appear after the vaccinated cohort reaches the high-risk ages (45 years and older) for cervical cancer—the cohort effect. This is why it takes a longer time to achieve the goal of cervical cancer elimination with an HPV vaccination coverage of 90% (scenario 3 in this study) compared to cytology- or HPV-based screening with a compliance of 70% (scenarios 1 and 2 in this study), reaching the goal in 2050 versus 2047 or 2035.

WHO announced the 90-70-90 target to achieve cervical cancer elimination: HPV vaccination coverage rate of 90% of girls by the age of 15 years, twice-lifetime screening of 70% of vaccinated women (by the age of 35 and 45 years), and treatment of 90% of women with the cervical disease [23]. The respective latest figures in Taiwan were 90% (HPV vaccination coverage rate; the actual figure could be higher because some women were vaccinated at their own expense and were not tallied), 43% (effective screening compliance rate), and 90% (proportion of treated for women who screened positive), with the effective screening compliance rate lagging far behind the 90-70-90 target. Our analysis shows that if the effective screening compliance rate can be increased to 70% in conjunction with an HPV coverage rate of 90%, Taiwan can achieve the goal of cervical cancer elimination in 2038 (scenario 4 in this study). However, it is worth noting that although the incidence rates of cervical cancer among all age groups have declined in Taiwan, there is a slightly increased trend in the 30-34 years age group among the most recent birth cohort (Figure S3 in Multimedia Appendix 1). This warrants attention from health authorities.

### Strengths and Limitations

The APC macrosimulation framework developed in this study is a useful policy analysis tool for disease control. The policy analysis results in this study can serve as a reference for other countries with a high incidence of cervical cancer. The strengths of this study lie in the rigorous analytical models we used and the high-quality data we used. Nonetheless, the study is not without limitations. First, we do not have access to individual-level data, and the study is therefore prone to ecological fallacy. Second, we used data spanning from 1997 to 2016 as the basis for making future predictions. This data range is unaffected by the delayed diagnosis and registration of cancer cases that occurred due to the COVID-19 pandemic [56]. Consequently, our short- to medium-term projections for cervical cancer incidence may be biased by the COVID-19 pandemic. Nonetheless, this is unlikely to affect the study’s primary conclusions, which focus on long-term projections. Finally, public funding for HPV vaccination in Taiwan is restricted to 13-year-old girls, leaving female individuals in other age groups the choice to opt for private vaccination. This study concentrates mainly on the consequences of the publicly funded HPV vaccination program and does not incorporate the likely effects of privately funded vaccinations in a wider age range. The high rate of HPV vaccination also fosters benefits through herd immunity [57], which could mean that our evaluations are underestimating the full potential impact of the vaccination program.

## Appendix

Multimedia Appendix 1 Additional data regarding incidence rate ratios, proportions of women, and cervical cancer incidence rates.

Multimedia Appendix 2 SAS code.

## Abbreviations

APC: age-period-cohort
HPV: human papillomavirus
PAF: population attributable fraction
SMAPE: symmetric mean absolute percentage error
WHO: World Health Organization

## Data Availability

The data sets generated and analyzed during this study are available from the corresponding author on reasonable request.
