# Supplementary material for: Projected Time for the Elimination of Cervical Cancer Under Various Intervention Scenarios: Age-Period-Cohort Macrosimulation Study
Source: JMIR Public Health Surveill. 2024 Apr 18;10:e46360. doi: 10.2196/46360 (PMC11066752; doi:10.2196/46360)
Supplement: Multimedia Appendix 1 [file publichealth_v10i1e46360_app1.pdf]

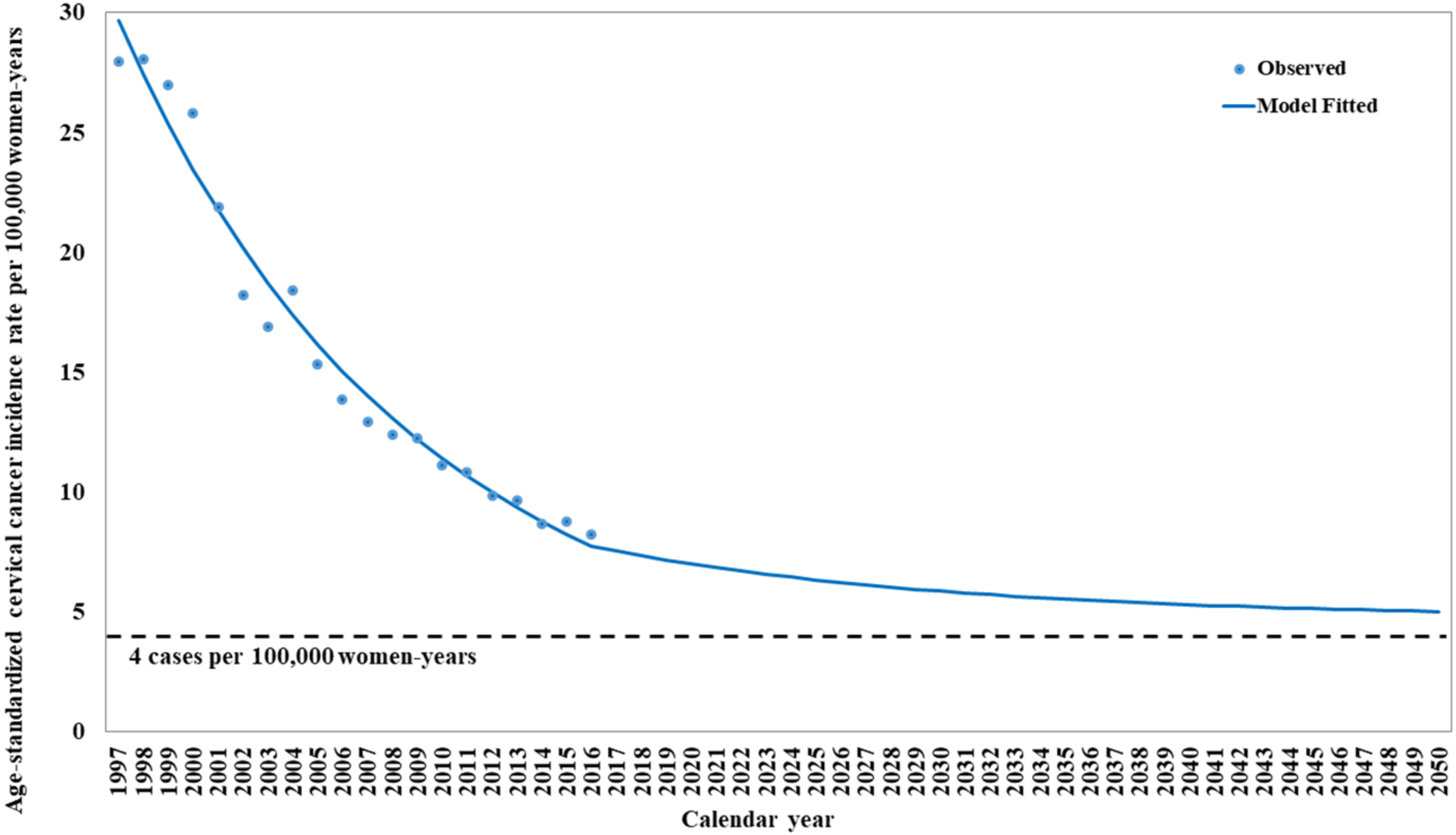

Figure S1. Age-standardized cervical cancer incidence rates from 1997 to 20106 and the projections from 2017 to 2050. The World Organization's 2000 World Standard Populations were used to compute the age-standardized incidence rate.

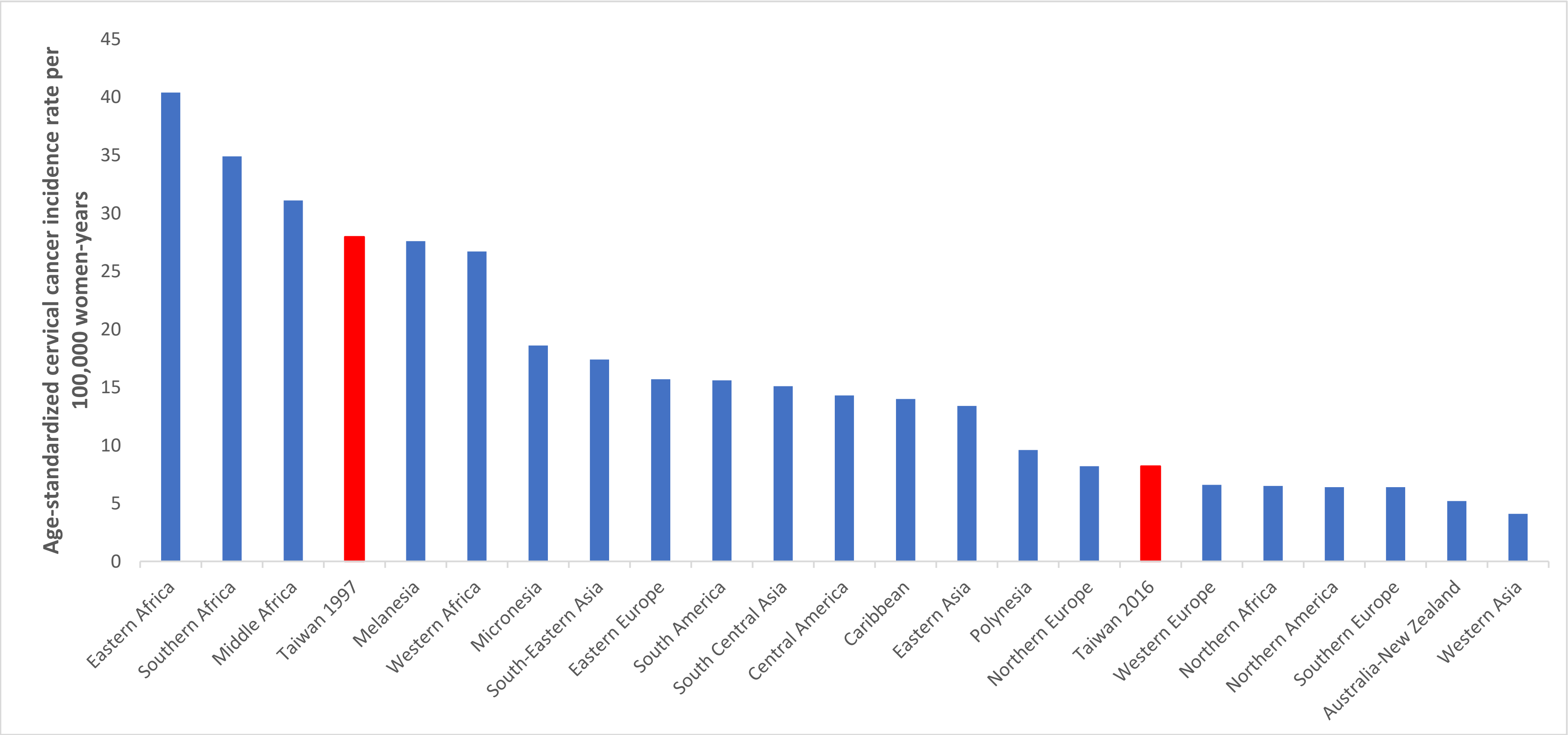

Figure S2. Global comparison of age-standardized cervical cancer incidence rates.
(Data from Global Cancer Statistics 2022:
Https://gco.iarc.who.int/today/en/dataviz/tables?mode=population&sexes=2&multiple_cancers=1&cancers=23&populations=905_906_910_911_912_913_914_915_916_920_921_922_923_924_925_926_927_928_931_954_957&multiple_populations=1&age_start=0)

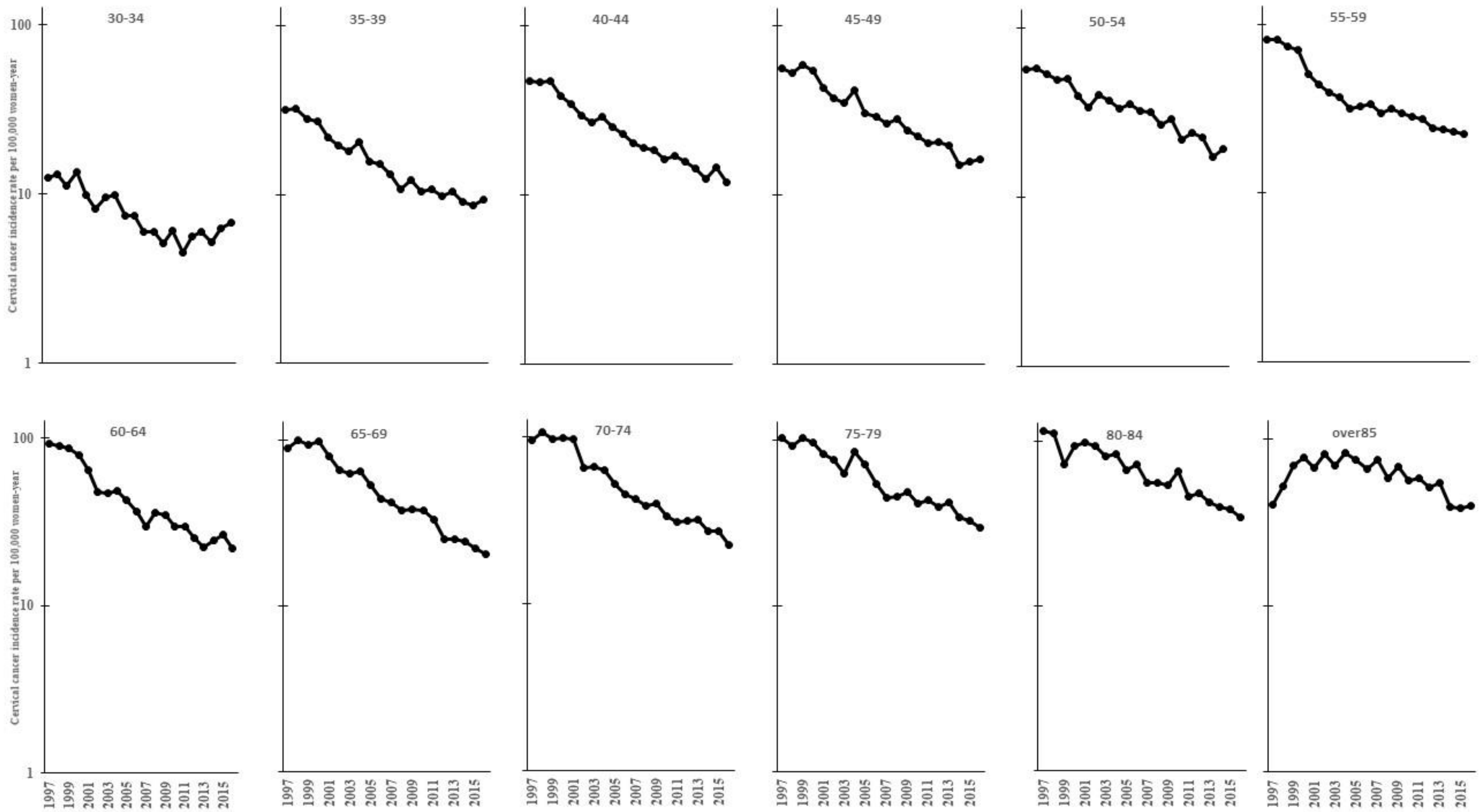

Figure S3. Age-specific cervical cancer incidence rates from 1997 to 2016.

Table S1. Incidence rate ratios of an integrated program involving both cytology-based screening/ HPV-based screening and HPV vaccination.

Frequency of cytology-based screening in 6 years.	Frequency of HPV-based screening in 6 years	HPV vaccination before 20 years old	Incidence rate ratio
0	0	No	1
1	0	No	0.581
2	0	No	0.432
3	0	No	0.325
≥4	0	No	0.262
0	0	Yes	0.120
1	0	Yes	0.581×0.120=0.070
2	0	Yes	0.432×0.120=0.052
3	0	Yes	0.325×0.120=0.039
≥4	0	Yes	0.262×0.120=0.031
0	0	No	1
0	1	No	0.432×0.6=0.259
0	0	Yes	0.120
0	1	Yes	0.432×0.6×0.120=0.031104

Table S2. Proportions of women in various levels of the screening/vaccination variable in 2016 in Taiwan.

Frequency of cytology-based screening in 6 years.	HPV vaccination before 20 years old	Proportion (%)
0	No	34.4
1	No	22.0
2	No	16.2
3	No	11.5
≥4	No	15.8
0	Yes	~ 0
1	Yes	~ 0
2	Yes	~ 0
3	Yes	~ 0
≥4	Yes	~ 0
