# Supplementary material for: Projected Time for the Elimination of Cervical Cancer Under Various Intervention Scenarios: Age-Period-Cohort Macrosimulation Study
Source: JMIR Public Health Surveill. 2024 Apr 18;10:e46360. doi: 10.2196/46360 (PMC11066752; doi:10.2196/46360)
Supplement: Multimedia Appendix 2 [file publichealth_v10i1e46360_app2.pdf]

```
/*SAS code*/

data apc;
input age period cases pop real; cards;
1 1 11 896260 11
2 1 33 903078 33
3 1 119 949945 119
4 1 295 931417 295
5 1 399 846357 399
6 1 368 658309 368
1 2 4 915479 4
2 2 30 896915 30
3 2 123 937744 123
4 2 305 947702 305
5 2 398 864956 398
6 2 379 712151 379
1 3 11 942297 11
2 3 30 886455 30
3 3 104 9320291 104
4 3 267 956555 267
5 3 418 886118 418
6 3 449 756695 449
1 4 6 975733 6
2 4 24 886144 24
3 4 90 917844 90
4 4 209 958232 209
5 4 314 913412 314
6 4 355 823681 355
1 5 . 976110 5
2 5 . 905514 19
3 5 . 911575 74
4 5 . 950255 185
5 5 . 927101 274
6 5 . 839730 313

1 6 . 971749 2
2 6 . 923362 21
3 6 . 905048 87
```

```
4 6 . 937006 168
5 6 . 941799 250
6 6 . 856895 300 ;
run;

%let I=6;  /*Age*/
%let J=4;  /*Period for training set*/
%let project=2;        /*Period for validation set */

data apc_poly; set apc2; age2=age1**2; age3=age1**3; age4=age1**4;
age5=age1**5;
period2=period1**2; period3=period1**3; cohort1=period1-age1+&I;
cohort2=cohort1**2; cohort3=cohort1**3; lnpop=log(pop);
pow2rate=(cases/pop)**(1/2); pow3rate=(cases/pop)**(1/3);
pow4rate=(cases/pop)**(1/4); pow5rate=(cases/pop)**(1/5); run;

proc sort data=apc_poly ; by age1;
run;

%MACRO create_knot_a(k);
%DO e=1 %TO &k;
knot_a&e=(round(age1-(&I/%eval(&k+1))*&e))**3; if knot_a&e<0 then knot_a&e=0;
%END;
%MEND;

%MACRO create_knot_p(k);
%DO e=1 %TO &k;
knot_p&e=(round(period1-(&J/%eval(&k+1))*&e))**3; if knot_p&e<0 then
knot_p&e=0;
%END;
%MEND;

%MACRO create_knot_c(k);
%DO e=1 %TO &k;
knot_c&e=(round(cohort1-((&I+&J-1)/%eval(&k+1))*&e))**3; if knot_c&e<0 then
knot_c&e=0;
%END;
%MEND;
```

```
%MACRO create_PRED(k);
%DO e=0 %TO &k %BY 5; pred&e=(pmark-pred0)*&e/100+pred0; %END;
%MEND;

%MACRO create_SMAPE(k,trans); if &trans=1 then do;
%DO e=0 %TO &k %BY 5;
mape&e=log(100*abs(pred&e-real/pop)/((abs(pred&e)+abs(real/pop))/2)
%END; end; else do;
%DO e=0 %TO &k %BY 5;
mape&e=(100*abs(pred&e-real/pop)/((abs(pred&e)+abs(real/pop))/2));
%END; end;
%MEND;

%MACRO create_logP(k, dist);
%DO e=0 %TO &k %BY 5;
if &dist='POISSON'
then logP&e=log(pdf('POISSON', real, pop*pred&e)); if &dist='NORMAL'
then logP&e=log(pdf('NORMAL',(real/pop)**pow,(pred&e)**pow, scale));
%END;
%MEND;

%MACRO create_vars(k);
%DO e=0 %TO &k %BY 5; mape&e logP&e %END;
%MEND;

%MACRO Select_APC(cov= , type= , res= , level= , trans= );
%LET vars=%create_vars(k=&level);

DATA input; SET apc_poly;
%LET K1=%SCAN(&res, 1, ' ');
%LET K2=%SCAN(&res, 2, ' ');
%LET K3=%SCAN(&res, 3, ' ');
%create_knot_a(k=&K1);
%create_knot_p(k=&K2);
%create_knot_c(k=&K3); RUN;

/*link=log*/
```

```
proc genmod data=input;
model cases=&cov /dist=poisson link=log offset=lnpop;
output out=poly(keep=age1 period1 real cases pop pred_cases) pred=pred_cases;
ods exclude all;
run;

proc iml; aa=&I; pp=&J; cc=aa+pp-1;
L_a=(1:aa)` - (aa/2 + 0.5);
L_p=(1:pp)` - (pp/2 + 0.5);
L_c=(1:cc)` - (cc/2 + 0.5); temp1=j(aa,1,1); temp2=L_a;
do i=2 to aa; temp1=temp1||temp2; temp2=temp2#L_a; end;
call gsorth(X_a,t,lin,temp1); temp1=j(pp,1,1); temp2=L_p;
do i=2 to pp; temp1=temp1||temp2; temp2=temp2#L_p; end;
call gsorth(X_p,t,lin,temp1); temp1=j(cc,1,1); temp2=L_c;
do i=2 to cc; temp1=temp1||temp2; temp2=temp2#L_c; end;
call gsorth(X_c,t,lin,temp1); X_a=(1:aa)`||X_a[,3:aa];
X_p=(1:pp)`||X_p[,3:pp];
X_c=(1:cc)`||X_c[,3:cc];
aname="a0":"a%eval(&I-2)";
pname="p0":"p%eval(&J-2)";
cname="c0":"c%eval(&I+&J-3)"; create X_a from X_a [colname=aname]; append
from X_a;
create X_p from X_p [colname=pname]; append from X_p;
create X_c from X_c [colname=cname]; append from X_c;
run; quit;

proc sort data=X_a out=X_a (rename=a0=age1); by a0;
run;

/*orthogonal age*/ data apc_poly; merge apc_poly X_a; by age1;
run;

proc sort data=apc_poly; by period1 age1;
run;


%Select_APC(cov=age1-age2 period1 period2 cohort2, type=Type1);
%Select_APC(cov=age1-age2 period1 cohort2, type=Type2);
```

```
%Select_APC(cov=age1-age3 period1-period3 cohort2 cohort3, type=Type3);
%Select_APC(cov=age1-age3 period1-period3 cohort2, type=Type4);
%Select_APC(cov=age1-age3 period1 period2 cohort2 cohort3, type=Type5);
%Select_APC(cov=age1-age3 period1 period2 cohort2, type=Type6);

data ppp(keep=age1 pmark); set poly; pmark=pred_cases/pop;
if period1=&J; run;

proc sort data=ppp; by age1; run; proc sort data=poly; by age1; run;
data attenuation; merge poly ppp; by age1; run;

proc sort data=attenuation; by period1 age1; run; data attenuation; set attenuation;
pred0=pred_cases/pop;
if period1<=&J then do;pmark=.;pred0=.;end;
%create_PRED(k=&level); run;

data mape; set attenuation;
if period1<=&J then do;pmark=.;pred0=.;end;
%create_SMAPE(k=&level,trans=&trans);
%create_logP(k=&level,dist='POISSON'); run;

proc means data=mape NOPRINT; var &vars;
output out=temp; run;

DATA TEMP; LENGTH ID $8; SET TEMP; ID='log'; RUN;

/*link=power 2*/
proc genmod data=input;
model pow2rate=&cov /dist=normal link=identity;
output out=poly(keep=age1 period1 real cases pop pred_cases) pred=pred_cases;
ods output ParameterEstimates=parms;
run;

DATA _NULL_; SET parms;
IF Parameter='scale' THEN DO; call symput('scale',Estimate); END; run; ods exclude
all;

data ppp(keep=age1 pmark); set poly; pmark=((pred_cases)**2);
```

```
if period1=&J; run;

proc sort data=ppp; by age1; run; proc sort data=poly; by age1; run; data
attenuation;
merge poly ppp; by age1; pred=((pred_cases)**2); run;

proc sort data=attenuation; by period1 age1; run; data attenuation; set attenuation;
pred0=((pred_cases)**2);
if period1<=&J then do;pmark=.;pred0=.;end;
%create_PRED(k=&level); run;

data mape; set attenuation;
if period1<=&J then do;pmark=.;pred0=.;end; scale=&scale; pow=1/2;
%create_SMAPE(k=&level,trans=&trans);
%create_logP(k=&level,dist='NORMAL'); run;

proc means data=mape NOPRINT; var &vars; output out=temp1; run;

DATA TEMP; SET TEMP temp1(in=A); IF A THEN ID='power2'; RUN;


%Select_APC(cov=age1-age3 period1 cohort2, type=Type7);
%Select_APC(cov=age1-age4 period1-period3 cohort2 cohort3, type=Type8);
%Select_APC(cov=age1-age4 period1 period2 period3 cohort2, type=Type9);
%Select_APC(cov=age1-age4 period1 period2 cohort2 cohort3, type=Type10);
%Select_APC(cov=age1-age4 period1 period2 cohort2, type=Type11);
%Select_APC(cov=age1-age4 period1 cohort2, type=Type12);
%Select_APC(cov=age1-age5 period1-period3 cohort2 cohort3, type=Type13);
%Select_APC(cov=age1-age5 period1-period3 cohort2, type=Type14);
%Select_APC(cov=age1-age5 period1 period2 cohort2 cohort3, type=Type15);
%Select_APC(cov=age1-age5 period1 period2 cohort2, type=Type16);
%Select_APC(cov=age1-age5 period1 cohort2, type=Type17);
%Select_APC(cov=age1 a1-a10 period1-period3 cohort2 cohort3, type=Type18);
%Select_APC(cov=age1 a1-a10 period1-period3 cohort2, type=Type19);
%Select_APC(cov=age1 a1-a10 period1 period2 cohort2 cohort3, type=Type20);
%Select_APC(cov=age1 a1-a10 period1 period2 cohort2, type=Type21);
%Select_APC(cov=age1 a1-a10 period1 cohort2, type=Type22);
%Select_APC(cov=age1-age3 knot_a1 knot_a2
```

```
period1-period3 knot_p1 knot_p2 cohort2 cohort3 knot_c1 knot_c2, type=Type23,
res=2 2 2);
%Select_APC(cov=age1-age3 knot_a1-knot_a3
period1-period3 knot_p1 knot_p2 cohort2 cohort3 knot_c1 knot_c2, type=Type24,
res=3 2 2);
%Select_APC(cov=age1-age3 knot_a1-knot_a4
period1-period3 knot_p1 knot_p2 cohort2 cohort3 knot_c1 knot_c2, type=Type25,
res=4 2 2);
%Select_APC(cov=age1-age3 knot_a1 knot_a2 period1-period3 knot_p1 knot_p2
cohort2 cohort3 knot_c1-knot_c3, type=Type26, res=2 2 3);
%Select_APC(cov=age1-age3 knot_a1-knot_a3 period1-period3 knot_p1 knot_p2
cohort2 cohort3 knot_c1-knot_c3, type=Type27, res=3 2 3);
%Select_APC(cov=age1-age3 knot_a1-knot_a4 period1-period3 knot_p1 knot_p2
cohort2 cohort3 knot_c1-knot_c3, type=Type28, res=4 2 3);
%Select_APC(cov=age1-age3 knot_a1 knot_a2 period1-period3 knot_p1 knot_p2
cohort2 cohort3 knot_c1-knot_c4, type=Type29, res=2 2 4);
%Select_APC(cov=age1-age3 knot_a1-knot_a3 period1-period3 knot_p1 knot_p2
cohort2 cohort3 knot_c1-knot_c4, type=Type30, res=3 2 4);
%Select_APC(cov=age1-age3 knot_a1-knot_a4 period1-period3 knot_p1 knot_p2
cohort2 cohort3 knot_c1-knot_c4, type=Type31, res=4 2 4);
%Select_APC(cov=age1-age3 knot_a1 knot_a2 period1-period3 knot_p1 knot_p2
cohort2 cohort3 knot_c1-knot_c5, type=Type32, res=2 2 5);
%Select_APC(cov=age1-age3 knot_a1-knot_a3 period1-period3 knot_p1 knot_p2
cohort2 cohort3 knot_c1-knot_c5, type=Type33, res=3 2 5);
%Select_APC(cov=age1-age3 knot_a1-knot_a4 period1-period3 knot_p1 knot_p2
cohort2 cohort3 knot_c1-knot_c5, type=Type34, res=4 2 5);
%Select_APC(cov=age1-age3 knot_a1 knot_a2 period1-period3 knot_p1 knot_p2
cohort2 cohort3 knot_c1-knot_c6, type=Type35, res=2 2 6);

/*link=power 3*/
proc genmod data=input;
model pow3rate=&cov/ dist=normal link=identity;
output out=poly(keep=age1 period1 real cases pop pred_cases) pred=pred_cases;
ods output ParameterEstimates=parms;
run;

DATA _NULL_; SET parms;
IF Parameter='scale' THEN DO; call symput('scale',Estimate); END; run; ods exclude
```

```
all;

data ppp(keep=age1 pmark); set poly; pmark=((pred_cases)**3);
if period1=&J; run;

proc sort data=ppp; by age1; run; proc sort data=poly; by age1; run; data
attenuation;
merge poly ppp; by age1; pred=((pred_cases)**3); run;

proc sort data=attenuation; by period1 age1; run; data attenuation; set attenuation;
pred0=((pred_cases)**3);
if period1<=&J then do;pmark=.;pred0=.;end;
%create_PRED(k=&level); run;

data mape; set attenuation;
if period1<=&J then do;pmark=.;pred0=.;end; scale=&scale; pow=1/3;
%create_SMAPE(k=&level,trans=&trans);
%create_logP(k=&level,dist='NORMAL'); run;

proc means data=mape NOPRINT; var &vars;
output out=temp1; run;

DATA TEMP; SET TEMP temp1(in=A); IF A THEN ID='power3'; RUN;

/*link=power 4*/
proc genmod data=input;
model pow4rate=&cov /dist=normal link=identity;
output out=poly(keep=age1 period1 real cases pop pred_cases) pred=pred_cases;
ods output ParameterEstimates=parms;
run;

DATA _NULL_; SET parms;
IF Parameter='scale' THEN DO; call symput('scale',Estimate); END; run; ods exclude
all;

data ppp(keep=age1 pmark); set poly; pmark=((pred_cases)**4);
if period1=&J; run;
```

```
proc sort data=ppp; by age1; run; proc sort data=poly; by age1; run; data
attenuation;
merge poly ppp; by age1; pred=((pred_cases)**4); run;

proc sort data=attenuation; by period1 age1; run; data attenuation; set attenuation;
pred0=((pred_cases)**4);

%Select_APC(cov=age1-age3 knot_a1-knot_a3 period1-period3 knot_p1 knot_p2
cohort2 cohort3 knot_c1-knot_c6, type=Type36, res=3 2 6);
%Select_APC(cov=age1-age3 knot_a1-knot_a4 period1-period3 knot_p1 knot_p2
cohort2 cohort3 knot_c1-knot_c6, type=Type37, res=4 2 6);
%Select_APC(cov=age1-age3 knot_a1 knot_a2
period1-period3 knot_p1-knot_p3 cohort2 cohort3 knot_c1 knot_c2, type=Type38,
res=2 3 2);
%Select_APC(cov=age1-age3 knot_a1-knot_a3
period1-period3 knot_p1-knot_p3 cohort2 cohort3 knot_c1 knot_c2, type=Type39,
res=3 3 2);
%Select_APC(cov=age1-age3 knot_a1-knot_a4
period1-period3 knot_p1-knot_p3 cohort2 cohort3 knot_c1 knot_c2, type=Type40,
res=4 3 2);
%Select_APC(cov=age1-age3 knot_a1 knot_a2 period1-period3 knot_p1-knot_p3
cohort2 cohort3 knot_c1-knot_c3, type=Type41, res=2 3 3);
%Select_APC(cov=age1-age3 knot_a1-knot_a3 period1-period3 knot_p1-knot_p3
cohort2 cohort3 knot_c1-knot_c3, type=Type42, res=3 3 3);
%Select_APC(cov=age1-age3 knot_a1-knot_a4 period1-period3 knot_p1-knot_p3
cohort2 cohort3 knot_c1-knot_c3, type=Type43, res=4 3 3);
%Select_APC(cov=age1-age3 knot_a1 knot_a2 period1-period3 knot_p1-knot_p3
cohort2 cohort3 knot_c1-knot_c4, type=Type44, res=2 3 4);
%Select_APC(cov=age1-age3 knot_a1-knot_a3 period1-period3 knot_p1-knot_p3
cohort2 cohort3 knot_c1-knot_c4, type=Type45, res=3 3 4);
%Select_APC(cov=age1-age3 knot_a1-knot_a4 period1-period3 knot_p1-knot_p3
cohort2 cohort3 knot_c1-knot_c4, type=Type46, res=4 3 4);
%Select_APC(cov=age1-age3 knot_a1 knot_a2 period1-period3 knot_p1-knot_p3
cohort2 cohort3 knot_c1-knot_c5, type=Type47, res=2 3 5);
%Select_APC(cov=age1-age3 knot_a1-knot_a3 period1-period3 knot_p1-knot_p3
cohort2 cohort3 knot_c1-knot_c5, type=Type48, res=3 3 5);
%Select_APC(cov=age1-age3 knot_a1-knot_a4 period1-period3 knot_p1-knot_p3
cohort2 cohort3 knot_c1-knot_c5, type=Type49, res=4 3 5);
```

```
%Select_APC(cov=age1-age3 knot_a1 knot_a2 period1-period3 knot_p1-knot_p3
cohort2 cohort3 knot_c1-knot_c6, type=Type50, res=2 3 6);
%Select_APC(cov=age1-age3 knot_a1-knot_a3 period1-period3 knot_p1-knot_p3
cohort2 cohort3 knot_c1-knot_c6, type=Type51, res=3 3 6);
%Select_APC(cov=age1-age3 knot_a1-knot_a4 period1-period3 knot_p1-knot_p3
cohort2 cohort3 knot_c1-knot_c6, type=Type52, res=4 3 6);

DATA result;
SET Type1-Type52;
MAPE=min(mape0, mape5, mape10, mape15, mape20, mape25, mape30, mape35,
mape40, mape45, mape50, mape55, mape60, mape65, mape70, mape75, mape80,
mape85, mape90, mape95, mape100);
logP=max(logP0, logP5, logP10, logP15, logP20, logP25, logP30, logP35, logP40,
logP45, logP50, logP55, logP60, logP65, logP70,logP75, logP80, logP85, logP90,
logP95, logP100);

PROC SORT; BY MAPE; RUN;


if period1<=&J then do;pmark=.;pred0=.;end;
%create_PRED(k=&level); run;

data mape; set attenuation;
if period1<=&J then do;pmark=.;pred0=.;end; scale=&scale; pow=1/4;
%create_SMAPE(k=&level,trans=&trans);
%create_logP(k=&level,dist='NORMAL'); run;

proc means data=mape NOPRINT; var &vars;
output out=temp1; run;

DATA TEMP; SET TEMP temp1(in=A); IF A THEN ID='power4'; RUN;

/*link=power 5*/
proc genmod data=input;
model pow5rate=&cov /dist=normal link=identity;
output out=poly(keep=age1 period1 real cases pop pred_cases) pred=pred_cases;
ods output ParameterEstimates=parms;
run;
```

```
DATA _NULL_; SET parms;
IF Parameter='scale' THEN DO; call symput('scale',Estimate); END; run; ods exclude
all;

data ppp(keep=age1 pmark); set poly; pmark=((pred_cases)**5);
if period1=&J; run;

proc sort data=ppp; by age1; run; proc sort data=poly; by age1; run; data
attenuation;
merge poly ppp; by age1; pred=((pred_cases)**5); run;

proc sort data=attenuation; by period1 age1; run; data attenuation; set attenuation;
pred0=((pred_cases)**5);
if period1<=&J then do;pmark=.;pred0=.;end;
%create_PRED(k=&level); run;

data mape;
set attenuation;
if period1<=&J then do;pmark=.;pred0=.;end; scale=&scale; pow=1/5;
%create_SMAPE(k=&level,trans=&trans);
%create_logP(k=&level,dist='NORMAL'); run;

proc means data=mape NOPRINT; var &vars;
output out=temp1; run;

DATA &type; LENGTH TYPE $10.;
SET TEMP temp1(in=A); TYPE="&type";
IF A THEN ID='power5'; IF _STAT_='MEAN';
DROP _FREQ_ _STAT_ _TYPE_ ; RUN;
%MEND;
```
